# Effect of a Cationic Surfactant on Microemulsion Globules and Drug Release from Hydrogel Contact Lenses

**DOI:** 10.3390/pharmaceutics11060262

**Published:** 2019-06-06

**Authors:** Cesar Torres-Luna, Naiping Hu, Abdollah Koolivand, Xin Fan, Yuli Zhu, Roman Domszy, Jeff Yang, Arthur Yang, Nam Sun Wang

**Affiliations:** 1 Department of Chemical & Biomolecular Engineering, University of Maryland, College Park, MD 20740, USA; cesartorres093@gmail.com (C.T.-L.); kolivand@umd.edu (A.K.); dwillyu@163.com (Y.Z.); 2 Lynthera Corporation, 1200 Corporate Blvd., STE 10C, Lancaster, PA 17601, USA; naiping.hu@gmail.com (N.H.); rcdom@istninc.com (R.D.); jeff@lynthera.com (J.Y.); 3 Department of Chemical Engineering, Auburn University, Auburn, AL 36849, USA; xzf0004@tigermail.auburn.edu

**Keywords:** drug delivery, microemulsion, contact lenses, cationic surfactant, controlled release

## Abstract

The present study evaluates the in vitro release of diclofenac sodium (DFNa) from contact lenses based on poly-2-hydroxyethyl methacrylate (pHEMA) hydrogels containing an embedded microemulsion to extend release duration. The oil (ethyl butyrate)-in-water microemulsion systems are prepared with two non-ionic surfactants, Brij 97 or Tween 80, together with a long-alkyl chain cationic surfactant, cetalkonium chloride (CKC). Without CKC, Brij 97 or Tween 80-based microemulsions showed average droplet sizes of 12 nm and 18 nm, respectively. The addition of CKC decreased the average droplet sizes to 2–5 nm for both non-ionic surfactants. Such significant reduction in the average droplet size corresponds to an increase in the DFNa release duration as revealed by the in vitro experiments. Contact lens characterization showed that important properties such as optical transparency and water content of Brij 97-based contact lenses with cationic microemulsions was excellent. However, the optical transparency of the corresponding Tween 80 based contact lenses was unsatisfactory. The results indicate that cationic microemulsion-laden contact lenses can benefit from combinatory effects of microemulsions and cationic surfactant at low CKC weight percentage, e.g., with the release of 70% of the drug in 45, 10, and 7 h for B97-CKC-0.45%, CKC-0.45%, and control lenses, respectively. However, the microemulsion effect on extending DFNa release became negligible at the highest CKC weight percentage (1.8%).

## 1. Introduction

Ophthalmic diseases are commonly treated with topically instilled eye drop solutions and suspensions. Such eye drop therapies are reported to comprise up to 90% of marketed formulations [1,2]. However, it has been estimated that eye drops have a bioavailability of less than 5% due to high tear turnover (blinking), induced lacrimation and nasolacrimal drainage of the solution, in addition to corneal and sclera permeation barriers [3,4]. 

The past 15 years have seen the re-emergence of research into the use of therapeutic contact lenses to increase the bioavailability of ocular drugs and improve patient compliance while minimizing systemic and ocular side effects [5,6,7]. Compared to eye drops, contact lenses reside in the tear film, which leads to a longer residence time and an optimum corneal delivery pathway for drug molecules [8]. However, a major limitation of contact lenses in drug delivery is that most of the drug absorbed in the contact lens is released within the first few hours which limits the use for extended release [9]. Several methods have been employed to increase the release duration of drugs, including nanoparticle-based contact lenses [10], biomimetic and imprinted contact lenses [11], layer-structured contact lenses [12], and lenses with vitamin E diffusion barriers [3,4,8,9,13,14]. A limited number of these approaches were examined in clinical trials such as the treatment of ocular allergy with ketotifen [15] and the treatment of glaucoma with timolol and dorzolamide [16].

Microemulsions offer a promising route for designing extended drug delivery systems due to improved drug solubilization, ease of preparation and administration, long shelf-life, improved corneal penetration and improved residence time [17,18,19,20]. Cationic emulsions increase the bioavailability of ophthalmic drugs in comparison to emulsions with no charge or negative charge [17,21,22]. The addition of cationic surfactant forms positively charged drops in microemulsions, which can bind to the negatively charged corneal epithelium, leading to increased residence time and drug permeability through the corneal epithelium [17]. Klang et al. [21] studied the influence of emulsion droplet surface charge on the distribution of indomethacin in ocular tissue, showing that the positive charge may prolong the residence time of the emulsion on the epithelial layer of the cornea and thus, enable better drug penetration through the cornea to the internal tissues of the eye. In addition, cationic emulsions have demonstrated to be effective and well-tolerated in animal studies for the treatment of corneal wounds and mild-to-moderate dry eye [23,24,25].

Due to the hydrophilic nature of pHEMA hydrogels, oil-in-water microemulsions embedded in pHEMA contact lenses are potential candidates as drug delivery vehicles. Contact lenses made with embedded microemulsions have been previously tested for extended drug delivery purposes [26,27,28,29,30]. Microemulsions can alter the tortuosity of pHEMA hydrogels [26] and hence, retard the drug transport of hydrophilic drugs such as timolol maleate [27] by alteration of the gel structure. In this case, drug molecules are forced to take a tortuous path to diffuse out from the lenses, resulting in a delayed release [3].

In this study, we investigate the effect of cetalkonium chloride (CKC) in oil-in-water microemulsions that are entrapped in pHEMA contact lenses, to enable controlled delivery of diclofenac sodium (DFNa). DFNa is an anionic NSAID for the treatment of ocular pain and eye inflammation. NSAIDs such as diclofenac and flurbiprofen have been previously tested in microemulsion formulations for sustained in vitro release [31,32,33]. CKC is a quaternary ammonium cationic surfactant that has been used in the development of stable oil-in-water nanoemulsions for topical ophthalmic drug delivery [22,34], having additional benefits in the protection and restoration of a healthy tear film and corneal epithelium [34]. Bengani et al. [35] utilized CKC to extend the drug delivery of the anionic drug dexamethasone 21-disodium phosphate from pHEMA contact lenses. Nevertheless, there are no reports of the application of cationic microemulsions for improved drug delivery from contact lenses. Therefore, we propose to use cationic microemulsions embedded in contact lenses that benefit from the combinatory effects of a microemulsion phase and a long chain cationic surfactant to modify the elution behavior of an anionic drug. In such systems, microemulsions serve as a diffusion barrier that retards DFNa release, while CKC further extends drug release due to the ionic interactions between the positively charged-contact lenses and the negatively charged drug.

## 2. Materials and Methods 

### 2.1. Materials

Diclofenac sodium (DFNa), ethyl butyrate (EB), Tween 80 (T80), Brij 97 (B97), cetalkonium chloride (CKC), 2-hydroxyethyl methacrylate (HEMA), ethylene glycol dimethacrylate (EGDMA), azobis-*iso*-butrylonitrile (AIBN) and Dulbecco’s phosphate buffer saline (PBS) were purchased from Millipore-Sigma (St. Louis, MO, USA). All chemicals were used as received.

### 2.2. Preparation of Microemulsions

A homogenous surfactant solution of surfactant (Brij 97 or Tween 80) and cationic surfactant (CKC) in 10 mL of deionized was obtain by stirring the mixture at 800 rpm for 4 h using a magnetic stir bar. This was followed by addition of ethyl butyrate to 5 mL of the aqueous surfactant solution with further mixing with a stir bar at 600 rpm for 20 min at 70 °C. A transparent microemulsion solution was obtained after the mixture was allowed to cool to room temperature. Table 1 summarizes the formulation compositions of the prepared microemulsion systems.

### 2.3. Characterization of Microemulsions

#### 2.3.1. Dynamic Light Scattering

The average globule size of microemulsions was determined with a Photocor Complex dynamic light scattering (DLS) instrument with Dynals analysis software (Photocor Ltd., Moscow, Russia). Before DLS measurement, we filtered the microemulsions through a 0.45 µm cellulose membrane. The readings were obtained at room temperature and at 90° scattering angle using a class IIIB laser with output at 633 nm/20 mW.

#### 2.3.2. Transmission Electron Microscopy (TEM)

We investigated the structural morphology of the microemulsions using transmission electron microscopy (JEOL JEM 2100 LaB6 high resolution TEM) with an accelerating voltage of 5 kV. For preparation of the samples for TEM imaging, a drop (~5 µL) of microemulsion was pipetted onto a holey carbon grid and a drop (~3 µL) of 3% ionic liquid (Hitachi HILEM IL1000™, C_7_H_19_NO_4_S) was pipetted onto the microemulsion immediately or within 2 min (i.e., before the sample dried). After 10 min, the excess ionic liquid was carefully absorbed/removed using a strip of filter paper. The sample was air dried overnight (~20 h) with a cover glass in place to prevent possible contamination. TEM images were captured using software (Digital Micrograph, Gatan, Inc., Pleasanton, CA, USA) via a CCD camera. Different magnifications were employed to study the size and morphology of the microemulsion oil droplets.

### 2.4. Fabrication of Microemulsion-Laden Contact Lenses 

To prepare the monomer mixture, 2 mL of HEMA was added to 1.5 mL of the EB-based microemulsion followed by 7.5 µL of EGDMA and 4.5 mg of AIBN with the ingredients mixed using a magnetic stir bar at 300 rpm for 10 min. EB was chosen as the oil phase due to its high solubility in HEMA-based aqueous solutions near equimolar composition [27]. The calculated weight percentages for CKC in the monomer mixture were approximately 0.45%, 1.8%, and 2.7% CKC for B97-CKC-0.45%, B97-CKC-1.8% and B97-CKC-2.7%, respectively. We utilized a plastic mold-casting method to fabricate the contact lenses (14 mm diameter, 8.4 mm base curve radius, and 120 µm thickness). Three drops of monomer solution (~75 µL) were added per each mold with a transfer-pipette and cured in an oven at 70 °C for 24 h. After curing, immersion of the molds in deionized water for 24 h removed unreacted monomer from the contact lenses. For the fabrication of control contact lenses, 1.5 mL of microemulsion was substituted with 1.5 mL of deionized water.

### 2.5. Fabrication of Cationic Surfactant-Loaded Contact Lenses (No Microemulsion)

Contact lenses with CKC only (without microemulsion) were also prepared. Firstly, after preparing a mixture of 2 mL of HEMA and 1.5 mL DI water we added 16, 63, 123, and 246 mg CKC, representing approximately 0.45, 1.8, 3.5, and 7.0% CKC weight percentage in the monomer mixture, respectively. Contact lenses were prepared following the same procedure as in Section 2.4 and we refer to the lenses as CKC-0.45%, CKC-1.8%, CKC-3.5%, and CKC-7.0%.

### 2.6. Drug Loading in Contact Lenses 

A 0.2 mg/mL diclofenac sodium in PBS solution was prepared for equilibrium loading in the fabricated contact lenses. Each air-dried contact lens was soaked in 3 mL of the diclofenac sodium solution for 7 days. To determine the amount of drug loaded in each lens, the drug concentration of each soaking solution was measured before and after the soaking period using a UV-visible spectrophotometer (Varian Cary 50 Bio, Walnut Creek, CA, USA) at a wavelength of 276 nm with pre-established calibration curves.

### 2.7. In Vitro Drug Release Experiments

After the drug-loading step, lenses were gently blotted to remove only excess drug solution from the contact lens surface. For release experiments, each contact lens was immersed in 3 mL of PBS at pH 7.4 and room temperature. At predetermined time intervals, 1 mL aliquots were pipetted out and replaced by 1 mL fresh PBS to simulate sink conditions. Drug concentration as a function of time was determined using the same procedure as in Section 2.6.

### 2.8. Characterization of Microemulsion-laden Contact Lenses

#### 2.8.1. Optical Transparency

To study how microemulsions affect the optical clarity of contact lenses, transmittance was measured using a UV-visible spectrophotometer (SpectraMax i3, Molecular Devices, Sunnyvale, CA, USA). Contact lenses were hydrated overnight by soaking in PBS at pH 7.4, and were placed in a plastic cuvette filled with 1 mL of PBS to measure transmittance. The transmittance at 630 nm was measured in triplicate.

#### 2.8.2. Water Content

The water content of contact lenses (hydrated for 24 h in simulated tear fluid at 23 °C) was determined using a gravimetric method. The hydrated contact lens was removed from the solution, gently blotted to remove all the liquid from the surface, and the wet weight was recorded. Dry weights were measured after removing the contact lenses from the hydrating solution, and letting them dry at room temperature overnight. The water content was measured in triplicate.

The water content is calculated by the lens weight percent increase from dry to wet:

$$\mathrm{Water}\;\mathrm{content}\;\left(\%\right)=\frac{{\mathrm{Weight}}_{\mathrm{wet}}-{\mathrm{Weight}}_{\mathrm{dry}}}{{\mathrm{Weight}}_{\mathrm{wet}}}\times 100$$

## 3. Results and Discussion

### 3.1. Characterization of Microemulsions

#### 3.1.1. Globule Size by Dynamic Light Scattering

To evaluate the effect of CKC on the oil droplet size, Brij 97 and Tween 80 microemulsions were prepared using two different cationic surfactant concentrations (weight %). As shown in Table 2, addition of cationic surfactant leads to a reduction in the average oil globule size.

We attribute this reduction in droplet size to the decrease in the surface tension of the surfactant solution due to the addition of CKC, which improves the dispersion of the oil phase in the surfactant solution. The enhanced dispersion of oil globules with smaller size may facilitate loading the drug more uniformly across the microemulsion-laden contact lenses.

#### 3.1.2. Globule Morphology by Transmission Electron Microscopy

**Figure 1:**
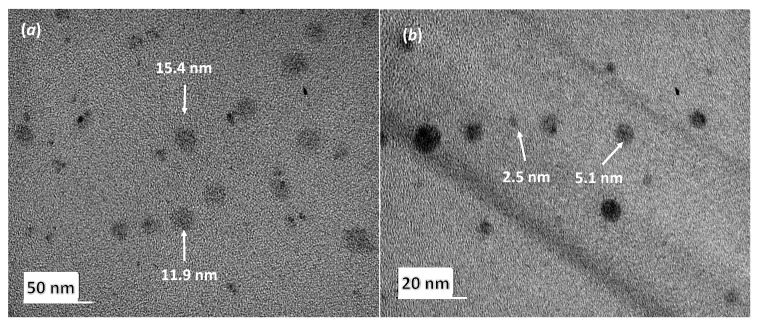
(**a**) TEM image of oil droplets in B97-CKC-0% microemulsion sample, and (**b**) TEM image of oil droplets in B97-CKC-1.8% microemulsion sample. TEM did not detect micelles in surfactant-only solutions.

Figure 1a shows the TEM image of the B97-CKC-0% microemulsion (microemulsions prepared without CKC). It shows spherical oil droplets between 11 nm and 16 nm size, which agrees with the results obtained by DLS and with a previous study that evaluated Brij 97-based microemulsions for the controlled delivery of cyclosporine [28].

Figure 1b shows the TEM image of a Brij 97-based microemulsion prepared with the presence of CKC (B97-CKC-1.8%). Figure 1b shows the spherical morphology of the oil droplets and reveals a droplet size ranging from 2 to 6 nm, and thus, confirms the reduced average droplet size measured by DLS for microemulsions with high CKC weight %.

### 3.2. In Vitro Drug Release

#### 3.2.1. Effect of Surfactant Weight % (B97) and Oil Weight % (EB) in Microemulsion

The octanol–water partition coefficient (log *P*) has been a common parameter to study the lipophilic character of drug compounds and the correlation of lipophilicity to pharmacokinetics and pharmacodynamics [36]. Diclofenac sodium has a log *P* of 4.75, and a low water solubility (DrugBank, ALOPGS source). Therefore, due to its relatively high log *P*, we expect DFNa to partition more in lipophilic phases than in aqueous phases.

**Figure 2:**
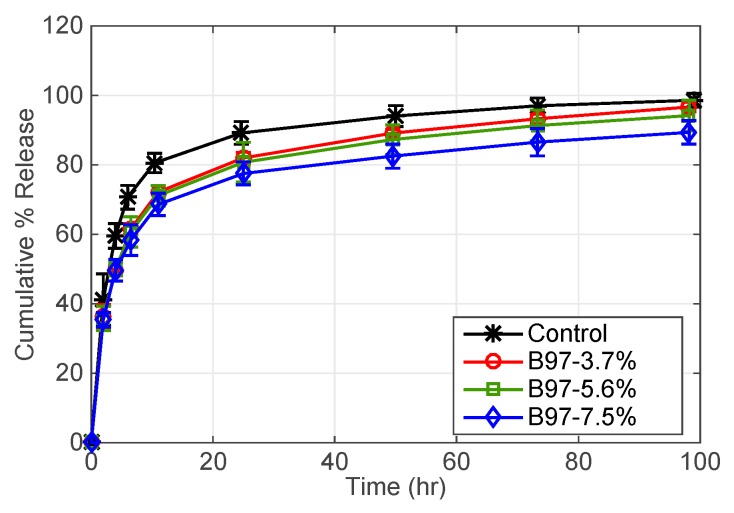
DFNa cumulative percent release as a function of time. Effect of B97 weight % in microemulsion laden-contact lenses. EB weight % is fixed at 0.8%. Amount of drug uptake (µg) described as follows: 132.0 ± 9.6 for control contact lenses; 157.7 ± 23.9 for B97-3.7%; 165.7 ± 3.5 for B97-5.6%; and 165.0 ± 8.7 for B97-7.5%. Data presented as mean ± SD (*n* = 3).

Figure 2 shows that the cumulative release of DFNa experiences a burst release followed by a nearly constant release after 20 h. Control contact lenses release 80% of loaded DFNa in 12 h, whereas DFNa release decreases to about 70% in the same time period with the addition of Brij 97. After 96 h, 90% of DFNa was released from B97-3.7% lenses, 88% of DFNa released from B97-5.6% lenses, and 80% of DFNa released from B97-7.5% lenses. Therefore, by increasing the weight % of Brij 97, the release of DFNa is not significantly extended.

**Figure 3:**
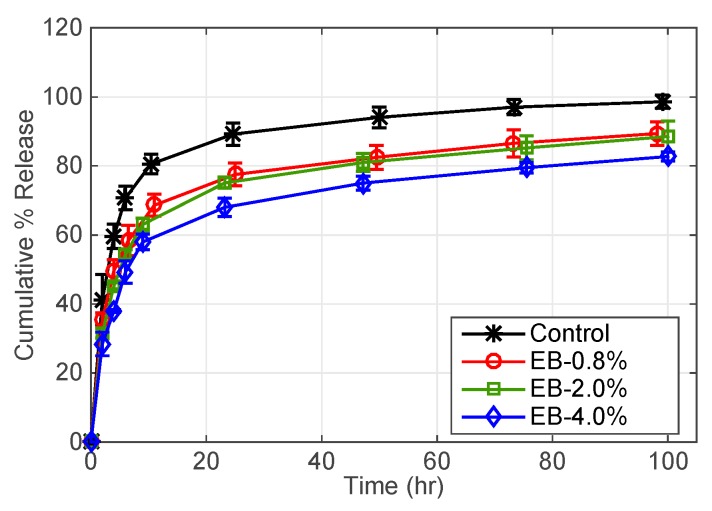
DFNa cumulative percent release as a function of time. Effect of EB oil weight % in microemulsion laden-contact lenses. B97 weight % is fixed at 7.5%. Amount of drug uptake (µg) described as follows: 132.0 ± 9.6 for control contact lenses; 165.0 ± 8.7 for EB-0.8%; 199.5 ± 10.5 for EB-2.0%; and 208.7 ± 17.9 for EB-4.0%. Data presented as mean ± SD (*n* = 3).

The cumulative release dependence on oil weight % is shown in Figure 3. As shown, increasing the oil phase by a factor of 2.5 does not have a significant effect in extending DFNa release; however, DFNa release was extended with an increase in the oil phase by a factor of 5. EB-0.8% contact lenses deliver 70% of DFNa after 12 h while EB-4% contact lenses deliver 70% of DFNa after approximately 40 h. The increase in the release time is because the oil phase acts as a diffusion barrier to retard DFNa release from contact lenses.

Although microemulsion-based contact lenses with a high oil concentration prolong the drug release duration, using high oil concentrations can impede or weaken the process of gel formation of contact lenses [27]. For the current study, we observed that EB-4% resulted in a non-transparent emulsion. The contact lenses made with EB-4% emulsions had acceptable optical transmission (>90%). However, the measured water content is below 30% (see Table 3). The decrease in the water content negatively impacts the oxygen permeability [37,38], and material modulus [39] of contact lenses. Therefore, the oil concentration at which the critical properties of contact lenses are not compromised should be established. Thus, the subsequent studies focus on contact lenses with the lowest oil concentration.

#### 3.2.2. Effect of Surfactant Type and Cationic Surfactant Weight % in Microemulsion

**Figure 4:**
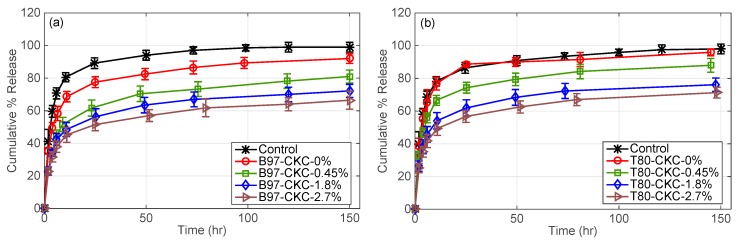
DFNa cumulative percent release as a function of time. Effect of CKC wt% in microemulsion laden-contact lenses. (**a**) Brij 97 surfactant and (**b**) Tween 80 surfactant. EB weight % is fixed at 0.8% and the non-ionic surfactants weight % are fixed at 7.5%. Amount of drug uptake (µg) described as follows: 132.0 ± 9.6 for control contact lenses; 165.0 ± 8.7 for B97-CKC-0%; 220.6 ± 24.0 for B97-CKC-0.45%; 235.9 ± 8.8 for B97-CKC-1.8%; 236.7 ± 12.5 for B97-CKC-2.7%; 186.2 ± 8.2 for T80-CKC-0%; 186.2 ± 8.2 for T80-CKC-0.45%; 218.5 ± 8.4 for T80-CKC-1.8%; and 223.8 ± 17.0 for T80-CKC-2.7%. Data presented as mean ± SD (*n* = 3).

In what follows, two different microemulsion systems based on Brij 97 or Tween 80 were studied in the presence of a cationic surfactant. Specifically, the effect of CKC and the type of non-ionic surfactant on DFNa release was studied. As shown in Figure 4, increasing the amount of CKC in microemulsion reduces the initial burst release and extends the release duration of DFNa for both microemulsion-laden contact lenses. Since DFNa is anionic at the physiological pH, it is hypothesized that electrostatic charges created by CKC increase adsorption of drug molecules to the hydrogel matrix, and hence minimize the burst release. Brij 97 and Tween 80 were both used as non-ionic surfactants and the effect of CKC occurred in both cases, validating the consistency of the obtained results.

Brij 97 and Tween 80 have been used in microemulsion systems for ocular drug delivery due to being safe and only weakly irritating to the eye [40]. The difference on the drug release kinetic between Brij 97-based and Tween 80-based contact lenses can be related to the physical properties of the non-ionic surfactants such as molecular weight, viscosity, and surface tension [32,40,41]. As shown in Figure 4, B97-CKC-0.45% contact lenses release 70% of DFNa in approximately 45 h while T80-CKC-0.45% lenses release 70% of DFNa in 20 h. Furthermore, B97-CKC-1.8% contact lenses release 70% of DFNa in 120 h, whereas T80-CKC-1.8% lenses release 70% of DFNa in 75 h. Therefore, contact lenses fabricated with Brij 97-microemulsions generate a slower release than lenses fabricated with Tween 80-microemulsions, and thus, they are more efficient to extend drug delivery.

#### 3.2.3. CKC-Microemulsion-laden and CKC-only Contact Lenses Comparison

**Figure 5:**
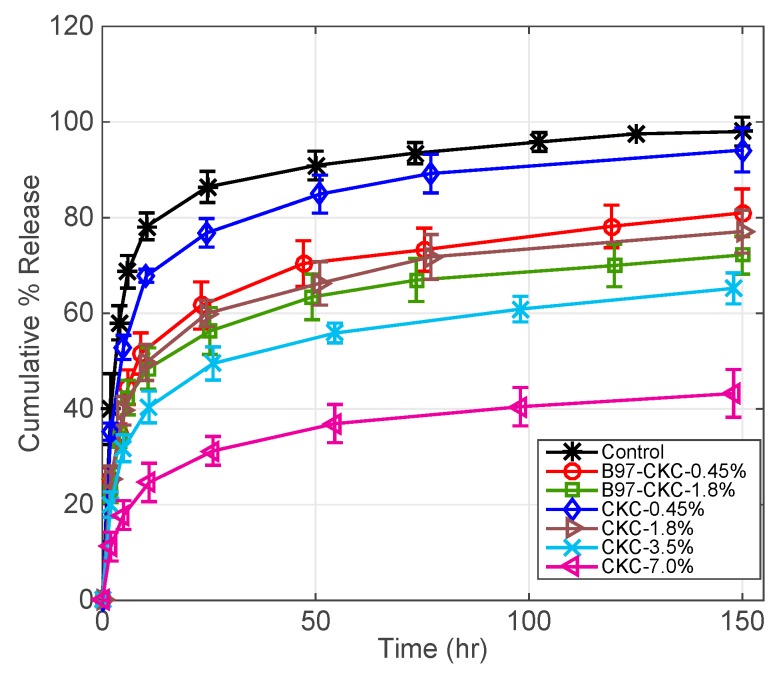
DFNa cumulative percent release as a function of time. Two types of contact lenses, CKC-only and CKC-microemulsion-laden contact lenses, with different CKC weight % are compared. EB weight % is fixed at 0.8% and the non-ionic surfactants weight % are fixed at 7.5%. Amount of drug uptake (µg) described as follows: 132.0 ± 9.6 for control contact lenses; 220.6 ± 24.0 for B97-CKC-0.45%; 235.9 ± 8.8 for B97-CKC-1.8%; 167.4 ± 6.5 for CKC-0.45%; 232.1 ± 17.0 for CKC-1.8%; 311.2 ± 19.8 for CKC-3.8%; and 443.1 ± 13.9 for CKC-7.6%. Data presented as mean ± SD (*n* = 3).

Figure 5 compares DFNa release profiles for contact lenses embedded with CKC-only and contact lenses combining microemulsion and CKC. Control contact lenses release 70% of DFNa in 7 h. Furthermore, B97-CKC-0.45% contact lenses released 70% of DFNa in approximately 45 h as mentioned in Section 3.2.2, while CKC-0.45% contact lenses released 70% of DFNa within the first 10 h. Furthermore, CKC-1.8% contact lenses released 70% of DFNa in 70 h while B97-CKC-1.8% contact lenses released 70% of DFNa in 120 h. For the same CKC weight percentages, the combined CKC-microemulsion system in contact lenses extends the release duration more than the CKC-only system. Therefore, even though the DFNa release is extended to a greater extent by CKC, the microemulsion phase still shows a noticeable effect on retarding DFNa release from contact lenses at relatively low CKC weight percentages. However, we expect that the effect of the microemulsion phase on extending drug release becomes negligible at CKC weight percentages above 1.8%.

For the case of CKC-3.5% and CKC-7.0%, the contact lenses released approximately 62% and 42% of DFNa within 100 h, respectively. The presence of CKC in the polymer matrix results in adsorption of the anionic DFNa on the charged-surfactant-coated surfaces, which reduces the diffusion rate and leads to extended drug release [35].

It should be noted that ocular toxicity studies on benzalkonium chloride (BKC), the parent molecule of CKC, suggest that high concentrations of BKC may cause ocular adverse effects on mitochondrial functionality and major cell loss or tissue alterations [42,43]. Thus, it is suggested that long-chain cationic CKC emulsions should be further developed in eye drops because of their reduced toxicity compared to BKC solutions [44]. CKC cationic nanoemulsions have shown to be safe and well tolerated following single and repeated applications, with neither inflammatory cell infiltration nor apoptosis [34]. In addition, CKC-based cationic nanoemulsions have been commercialized since 2008 (Cationorm), and the use of CKC as cationic surfactant in emulsions is now protected by several granted and pending European and US patents [22,34]. Due to their high lipophilicity, long-alkyl chain cationic surfactants loaded on contact lenses can minimize the potential release of the surfactants into the tears after the contact lens is inserted in the eyes [34,35]. Nevertheless, in vitro studies on a corneal cell and/or in vivo studies in large animals are still required to assess the efficacy of these biomaterials.

### 3.3. Characterization of Contact Lenses

#### 3.3.1. Optical Transmission

Table 3 describes the % transmittance of the contact lenses studied in the in vitro release experiments. The reduction in the % transmittance is an indication of larger average oil globule size and destabilization of the microemulsions, while an increase in the % transmittance indicates a smaller average oil globule size and a stable microemulsion [30]. An optical clarity above 92% is considered as an acceptable commercial optical clarity value [45]. It is observed that all Brij 97-based microemulsion contact lenses have optical transmissions above 92%. For Tween 80-based microemulsions-laden contact lenses, transmission significantly increases from 30% to 85% by increasing the CKC weight % in microemulsions from 0 to 1.8%, respectively.

The reduced transmittance of Tween 80 lenses is due to its high solubility in the HEMA monomer. Hence, upon mixing the monomer with the microemulsion, the surfactant molecules desorbed from the oil drops, causing destabilization and aggregation of oil drops [26].

Maulvi et al. [29,30] concluded that the drug release from microemulsion-laden contact lenses is sustained to a greater extent when the stability of the microemulsion is improved. The microemulsion stability was improved by utilizing surfactants with long carbon chain lengths [29,30]. Moreover, the % transmittance of a microemulsion-based contact lens was increased when the stable microemulsion was achieved [30]. Based on our results, the addition of CKC in microemulsions extended the release of DFNa for both Brij 97 and Tween 80-based microemulsion contact lenses. The extension of DFNa release was directly proportional to the weight percentage of CKC in the microemulsions. Furthermore, the inclusion of CKC improves the optical transmission of Tween 80-based contact lenses and retains the high optical transmission of Brij 97-based lenses. Therefore, based on the results, we tentatively conclude that the CKC-microemulsions were stable in the contact lenses within the time frame in which the release studies were performed. However, additional stability experiments would need to evaluate the long-term stability of these cationic microemulsions in the contact lenses.

#### 3.3.2. Water Content

From Table 3, the water content does not change by more than 10% with respect to the control contact lenses (i.e., pure pHEMA), except for EB-4% contact lenses. For Brij 97-based microemulsion contact lenses, water content varies between 36.9% and 39.2%, while for Tween 80-based microemulsion contact lenses, it is varying from 33.1% to 39.7%. Furthermore, water content does not significantly change for CKC-3.5% and CKC-7.0% contact lenses.

## 4. Conclusions

Previous studies developed oil-in-water microemulsion systems for pHEMA contact lenses, e.g., studies in References [26,27,28,30] that extended the release duration of ophthalmic drugs. Other studies utilized cationic surfactants to extend the release of anionic drugs in pHEMA contact lenses, e.g., study in Reference [35]. The present work incorporated the microemulsion approach with a cationic surfactant in the fabrication of contact lenses and demonstrated the extended release of DFNa from CKC-microemulsion embedded contact lenses. Microemulsions made with low oil weight % are transparent. Although an emulsion created by high oil weight % can be embedded in contact lenses to extend the release kinetics, it carries the risk of impacting the critical properties. We used two non-ionic surfactants, Brij 97 or Tween 80, to prepare the microemulsions. The increase in the DFNa release time along with the negligible effects on the optical transmission and water content of the lenses suggest that Brij 97 is a better surfactant for the fabrication of CKC-microemulsion contact lenses. Optical transmission of Tween 80-based contact lenses was generally compromised at low loading of CKC (weight %) due to its high solubility in HEMA-based aqueous solutions. The CKC-microemulsion contact lenses extended the DFNa release time by a greater extent than the CKC-only contact lenses at low CKC weight percentage, i.e., 0.45%. However, by increasing the CKC concentration to 1.8%, we observed that the microemulsion began to play a minimal role on retarding drug release. The CKC-microemulsions were stable in the contact lenses over the time period of the release studies. However, future works need to further evaluate the critical properties of contact lenses, such as material modulus, oxygen permeability and wettability as well as cytotoxicity and animal studies to verify the contact lenses safety, efficacy, toxicity, and long-term stability before commercialization.

## Figures and Tables

**Table 1:** Summary of the microemulsions systems.

Formulation	Oil	Surfactant		Cationic Surfactant
	EB (µL)	Brij 97 (g)	Tween 80 (g)	CKC (mg)
B97-3.7%	100	1	-	-
B97-5.6%	100	1.5	-	-
B97-7.5% *	100	2	-	-
EB-2% **	250	2	-	-
EB-4% **	500	2	-	-
B97-CKC-0.45%	100	2	-	125
B97-CKC -1.8%	100	2	-	500
B97-CKC -2.7%	100	2	-	750
T80-CKC-0%	100	-	2	-
T80-CKC-0.45%	100	-	2	125
T80-CKC-1.8%	100	-	2	500
T80-CKC-2.7%	100	-	2	750

* We refer to B97-7.5% microemulsions as EB-0.8% in Figure 3 (effect of oil weight % on release kinetics), and as B97-CKC-0% in Figure 4a (effect of cationic surfactant weight % on release kinetics) and Table 2 to facilitate the comparison. ** Emulsions not transparent (opaque) after equilibration.

**Table 2:** Average oil globule size obtained from each microemulsion.

Formulation	Average Oil Globule Size (nm)
B97-CKC-0%	12.1 ± 1.8
B97-CKC-0.45%	3.4 ± 0.3
B97-CKC-1.8%	2.4 ± 0.2
T80-CKC-0%	18.1 ± 3.2
T80-CKC-0.45%	5.0 ± 0.4
T80-CKC-1.8%	2.7 ± 0.6

**Table 3:** Characterization of contact lenses used for in vitro release studies. Data are presented as mean ± SD with *n* = 3.

Formulation	Transmittance (%)	Water Content (%)
Control	99.5 ± 0.4	36.7 ± 4.8
EB-4%	94.7 ± 4.9	27.6 ± 4.1
T80-CKC-0%	30.8 ± 5.3	33.1 ± 5.8
T80-CKC-0.45%	43.8 ± 5.8	39.7 ± 1.4
T80-CKC-1.8%	85.7 ± 7.1	34.8 ± 1.6
B97-CKC-0%	95.5 ± 2.2	39.2 ± 0.7
B97-CKC-0.45%	94.7 ± 4.6	38.8 ± 0.5
B97-CKC-1.8%	95.2 ± 4.0	36.9 ± 2.3
CKC-3.5%	99.1 ± 0.4	33.9 ± 5.0
CKC-7.0%	99.5 ± 0.2	33.5 ± 1.5
